# Life cycle assessment for early-stage process optimization of microbial biosurfactant production using kinetic models—a case study on mannosylerythritol lipids (MEL)

**DOI:** 10.3389/fbioe.2024.1347452

**Published:** 2024-02-23

**Authors:** Lars Bippus, Ann-Kathrin Briem, Alexander Beck, Susanne Zibek, Stefan Albrecht

**Affiliations:** ^1^ Department Life Cycle Engineering GaBi, Institute for Acoustics and Building Physics IABP, University of Stuttgart, Stuttgart, Germany; ^2^ Department Life Cycle Engineering GaBi, Fraunhofer Institute for Building Physics IBP, Stuttgart, Germany; ^3^ Fraunhofer Institute for Interfacial Engineering and Biotechnology IGB, Stuttgart, Germany; ^4^ Institute for Interfacial Process Engineering and Plasma Technology IGVP, University of Stuttgart, Stuttgart, Germany

**Keywords:** biosurfactant, mannosylerythritol lipids (MELs), life cycle assessment (LCA), fermentation, downstream processing, biotechnology, prospective LCA

## Abstract

**Introduction:** This study assesses the environmental impacts of mannosylerythritol lipids (MELs) production for process optimization using life cycle assessment (LCA). MELs are glycolipid-type microbial biosurfactants with many possible applications based on their surface-active properties. They are generally produced by fungi from the family of Ustilaginaceae via fermentation in aerated bioreactors. The aim of our work is to accompany the development of biotechnological products at an early stage to enable environmentally sustainable process optimization.

**Methods:** This is done by identifying hotspots and potentials for improvement based on a reliable quantification of the environmental impacts. The production processes of MELs are evaluated in a cradle-to-gate approach using the Environmental Footprint (EF) 3.1 impact assessment method. The LCA model is based on upscaled experimental data for the fermentation and purification, assuming the production at a 10 m³ scale. In the case analyzed, MELs are produced from rapeseed oil and glucose, and purified by separation, solvent extraction, and chromatography.

**Results:** The results of the LCA show that the provision of substrates is a major source of environmental impacts and accounts for 20% of the impacts on Climate Change and more than 70% in the categories Acidification and Eutrophication. Moreover, 33% of the impacts on Climate Change is caused by the energy requirements for aeration of the bioreactor, while purification accounts for 42% of the impacts respectively. For the purification, solvents are identified as the main contributors in most impact categories.

**Discussion:** The results illustrate the potentials for process optimization to reduce the environmental impacts of substrate requirements, enhanced bioreactor aeration, and efficient solvent use in downstream processing. By a scenario analysis, considering both experimental adaptations and prospective variations of the process, the laboratory development can be supported with further findings and hence efficiently optimized towards environmental sustainability. Moreover, the presentation of kinetic LCA results over the fermentation duration shows a novel way of calculating and visualizing results that corresponds to the way of thinking of process engineers using established environmental indicators and a detailed system analysis. Altogether, this LCA study supports and demonstrates the potential for further improvements towards more environmentally friendly produced surfactants.

## Introduction

### Motivation and background

The production of sustainable and low impact products is essential, in order to face environmental challenges, such as climate change. The European Commission is committed to achieving climate neutrality by 2050 and has therefore presented the European Green Deal (European Commission, 2019), with the European Bioeconomy strategy contributing to these ambitious plans (European Commission, 2012). Biosurfactants can contribute to achieve these aims, due the use of exclusively biobased raw materials for their production. While traditional surfactants are synthesized chemically from either petrochemical or oleochemical resources, the term biosurfactant usually refers to surfactants that are produced biotechnologically, either by enzymatic or microbial synthesis from renewable resources. Microbial biosurfactants, which are produced by fermentation of bacteria or fungi in a bioreactor, are commonly divided into high molecular weight and low molecular weight biosurfactants, with the former including lipoproteins and lipopolysaccharides, and the latter including lipopeptides or glycolipids. Prominent examples of microbial biosurfactants are the glycolipid-type rhamnolipids and sophorolipids or the lipopeptide surfactin (Sarubbo et al., 2022).

Biosurfactants share many properties with their synthetic counterparts, such as surface tension reduction, foaming, wetting, emulsification, and phase formation, but the specific properties of a particular class of biosurfactant always depend on its chemical structure. They are readily biodegradable, due to their exclusively bio-based raw materials and catalysts (Klosowska-Chomiczewska et al., 2011; Rodríguez-López et al., 2020; Briem et al., 2022). In addition, biosurfactants offer further advantages for environmentally friendly surfactant production, such as low reaction temperatures, due to their microbial or enzymatic synthesis. The variety of structures and functional properties of microbial biosurfactants allow for a wide range of possible applications for these surface-active agents, for example, their use in detergents and cleaners, in the food industry, cosmetics, medicine and pharmaceuticals, nanotechnology, agriculture or bioremediation (Marchant and Banat, 2012b; Kosaric and Vardar-Sukan, 2015; Hayes et al., 2019; Sarubbo et al., 2022; Zibek and Soberón-Chávez, 2022).

As such, microbial biosurfactants could contribute to a functioning bioeconomy, although their superior sustainability over established chemical surfactants, whose production routes have often been optimized for a long time, still needs to be proven (Marchant and Banat, 2012a). In order to make a reliable statement regarding product sustainability, it is thus necessary to assess several sustainability aspects using a systematic assessment method. LCA thus serves as a method for systematically determining and evaluating the environmental sustainability aspects based on mass and energy balances. The European Platform on LCA (EPLCA) promotes LCA as an essential integrated environmental assessment method to support the goals of the European Green Deal and the EU policy making process (European Commission, 2019), including various initiatives and programs, such as the Circular Economy Action Plan, the Farm to Fork strategy, the Biodiversity strategy, the Chemical strategy, and many more (European Union, 2023). Consequently, only if biosurfactants offer measurable environmental benefits are they preferable to established conventional or petrochemical-based products. LCA studies on biosurfactant production can therefore contribute to understanding their essential environmental sustainability aspects and to obtain a clearer picture of their product sustainability. Although the benefits of life cycle assessments are obvious and they are widely accepted and applied in R&D, industry and politics, there are hardly any complete and comprehensive life cycle assessments for biosurfactants to be found in the literature (Briem et al., 2022).

### Production and properties of mannosylerythritol lipids

**FIGURE 1:**
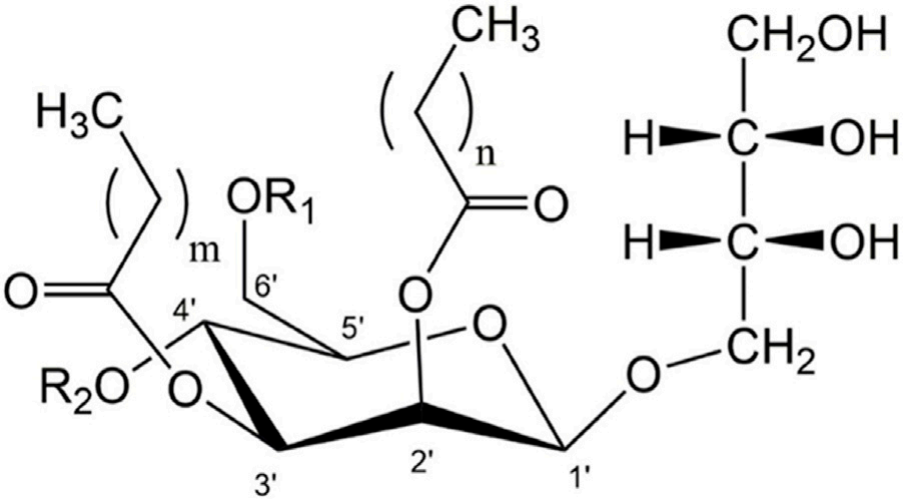
General structure of di-acylated mannosylerythritol lipids (MEL). The different variants MEL-A, -B, -C and -D (MEL-A: R1 = R2 = Ac; MEL-B: R1 = Ac, R2 = OH; MEL-C: R1 = OH, R2 = Ac, MEL-D: R1 = R2 = OH) with varying length of fatty acid side-chains at C2’ and C3’ (m = 2–16, n = 2–10) depend on the microorganism used for their production (Beck et al., 2019a).

A particularly interesting class of microbial biosurfactants are mannosylerythritol lipids (MELs). MELs are microbially produced non-ionic surfactants and are classified as glycolipid-type microbial biosurfactants. They contain a polar 4-O-β-d-mannopyranosyl-d-erythritol head group and several lipophilic fatty acid groups. The most common MELs have two fatty acid chains at C2’ and C3’ of the mannose moiety and a variable degree of acetylation at C4’ and C6’ (Figure 1). Variations in the position, number, chain length and degree of saturation of the fatty acid groups affect the chemical structure and therefore the properties of the surfactants. Like all microbial biosurfactants, MELs are produced as a mixture of the different congeners, the composition of which is mostly strain-specific (Beck et al., 2019a). By using different microorganisms and substrates, surfactant properties can be tuned and optimized for a specific application. The microbial species used for MEL production belong to genera such as *Moesziomyces* or *Ustilago* sp. within the family of Ustilaginaceae fungi. The most effective substrates for MEL production are vegetable oil, such as soybean, rapeseed or olive oil. Glucose or other sugars can also be used to promote microbial growth, but the addition of vegetable oils is always necessary to achieve high MEL concentrations. The production process has so far been demonstrated in many shake flask cultivations (for an overview see (Beck et al., 2019b), but also in some bioreactor studies (Rau et al., 2005; Kim et al., 2006; Goossens et al., 2016; Beck et al., 2022).

The special structure of MEL biosurfactants leads to a variety of interesting properties and possible applications. As surfactants, they reduce the surface tension of water to about 24–31 mN/m at a critical micelle concentration of 2.7–6.0 × 10^−6^ mol/L, depending on the type of MEL (Morita et al., 2015). Their phase behavior and self-assembly properties have been studied in detail (Kitamoto et al., 2009). In addition, MELs have been shown to induce cell differentiation in mammalian cells (Isoda et al., 1997; Wakamatsu et al., 2001), to interact with proteins (Kitamoto et al., 2000; Konishi et al., 2007), and even to have anti-microbial activity against Gram-positive bacteria (Kitamoto et al., 1993; Shu et al., 2020). Due to their surface-wetting ability, they have been proposed as agrochemicals (Fukuoka et al., 2015). Perhaps the most interesting application is their use in cosmetics and personal care, where they can moisturize and repair damaged hair and skin (Morita et al., 2009; Morita et al., 2010).

### State of the art of LCA on biosurfactants

Implementing LCA-based process optimization in an early product development stage allows to minimize later efforts for extensive adaptations of an industrial process to achieve an overall environmentally friendly process. This way, process optimization can improve the environmental performance of a product more easily than implementing environmental protection measures in the mature process. This applies to the production of MEL, as these are currently being produced on a pilot scale and the upscaling is currently under development. For this reason, environmental hotspots can be identified with tools, such as LCA, and a scenario analysis highlights potentials for optimizations and serves as a decision-making tool to prioritize optimization measures.

In 2013, the European Commission started to develop the Product Environmental Footprint Category Rules (PEFCR) for shampoos to establish the basis for comparable life cycle assessment within this product category (Golsteijn et al., 2015). This presented the challenge of providing sufficiently reliable and representative life cycle inventory (LCI) data for the production of surfactants to achieve adequate comparability. A study by ERASM (Schowanek et al., 2018) provides well-founded LCA data for a number of widely used conventional surfactants. Some of these surfactants are produced entirely or partly from biobased precursors, however no biotechnologically produced surfactants are considered in this study.

A recent literature review on LCA of microbial biosurfactants found that there is limited availability of complete and comprehensive LCA studies on biosurfactants publicly available and, therefore, research needs were identified in this field (Briem et al., 2022). The studies available differ in biosurfactant type, production scales and raw material input, but also in the definition of goal and scope, system boundaries, geographical reference, and impact assessment methods (Briem et al., 2022). For these reasons, the results of these studies are often not comparable. Besides three studies on alkyl polyglycosides (APGs) (Guilbot et al., 2013; Brière et al., 2018; Lokesh et al., 2019), which are counted as biosurfactants depending on the definition as mentioned above, only a very limited number of published studies on microbially produced biosurfactants were identified. Baccile et al. (2017) assessed the environmental impacts of a sophorolipid in a hand washing application. They used upscaled data from a pilot plant for the production phase, however the whole product life cycle is included in their LCA study. Aru and Onwurah Ikechukwu, (2018) discussed the impacts of a biosurfactant, which is not further specified. Kopsahelis et al. (2018) performed a cradle-to-gate LCA of sophorolipids and rhamnolipids from waste oil and sugar, based on data from a pilot plant. Balina et al. (2023) conducted a prospective cradle-to-gate LCA for the production of sophorolipids to identify potentials for process optimization, while Schonhoff et al. (2022) performed an LCA on rhamnolipids and MELs production from different sugar substrates. Despite the limited number of studies, the authors of the literature review on biosurfactants concluded that the main influence on the environmental impacts in the production phase of the biosurfactants is due to the raw material production and the energy demand for the fermentation processes.

Well-founded life cycle assessments with a robust database provide helpful and meaningful information for optimizing production processes and contribute to making biosurfactant production environmentally sustainable. This is where this work goes beyond the state of the art of LCA on biosurfactants and addresses these needs, offering a much more detailed look at the environmental aspects of production and identifying optimization potentials that bioprocess developers can use to optimize their processes and products’ sustainability.

In order to obtain an understanding of the environmental aspects of bioprocesses using the case of MEL production, the performed LCA study with scenario analysis highlights potential improvements for the MEL production. It can, thus, support the development work of process designers. Moreover, by combining the LCA model with a kinetic model, an innovative approach was developed to determine the environmental optimum for the process duration from an environmental point of view. This novel approach and type of analysis gains new insights and supplements static LCA results.

## Materials and methods

LCA is an internationally recognized scientific method and environmental management tool used to evaluate the environmental impacts of a product or service based on mass and energy balances. For this purpose, material and energy flows including use of resources and emissions are assessed along the product´s life cycle following a standardized approach according to ISO 14040 and ISO 14044 (ISO 14040:2006, 2006a; ISO 14044:2006, 2006b). LCA can be used as a decision-making tool to identify environmental hotspots in an early design stage and help to optimize product design to enhance the environmental performance of the investigated system.

### Goal of the study, system boundary and functional unit

**FIGURE 2:**
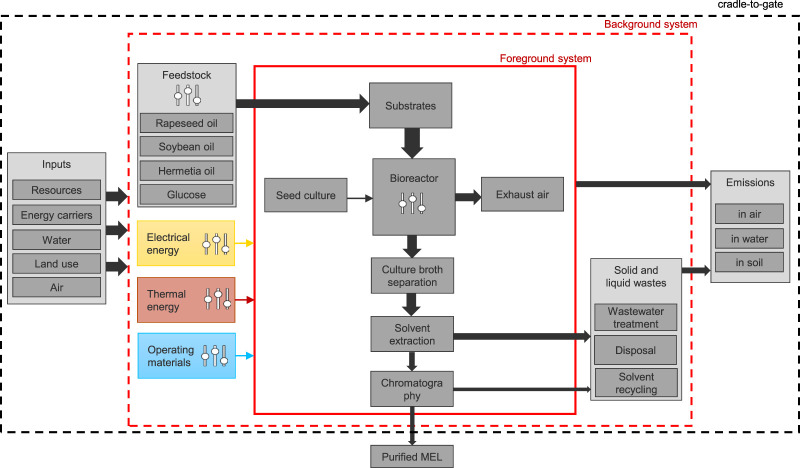
Process flow chart for MEL fermentation and purification with system boundaries.

The LCA performed in this study aims to identify relevant process steps and raw materials in the production and downstream processing (DSP) of MEL for overall process optimization. Based on the results of the LCA, hotspots can be identified so that priorities and measures for process optimizations can be derived to improve the environmental performance of the overall production process. The system analyzed consists of the provision of raw materials (substrates), the fermentation in the bioreactor and the necessary purification steps for the production of the pure MEL biosurfactant. A process flowchart of the MEL production is presented in Figure 2.

At present, no reliable assumption can be made about the use phase of the biosurfactant, due to the early stage of development and the wide range of potential areas of application. The use strongly depends on the properties, and research on future applications and formulations of final products need to be carried out to make well-founded assumptions about the use and disposal phase. Accordingly, the functional unit for the goal of this study was defined as “1 kg of pure MEL” and the system boundaries were defined as cradle-to-gate. This includes the production processes for MEL with fermentation and downstream processing respectively, including all upstream chains from the mining and cultivation of raw materials, the production of intermediate products, such as substrates, operating materials, and energy supply.

### Inventory and data collection

**FIGURE 3:**
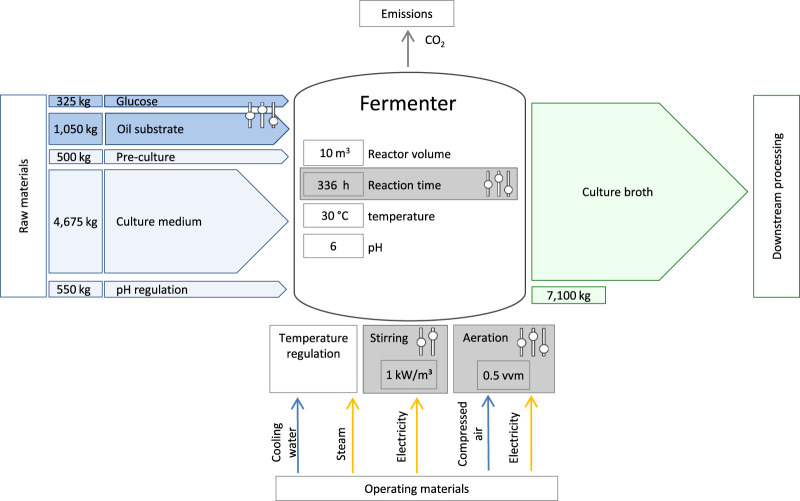
Schematic illustration of the bioreactor used for MEL production with main material flows representing the base case (scenario 3) and the process parameters that were varied during the scenario analysis.

The production system is based on the prospective upscaling of experimental data from a laboratory scale bioreactor. A detailed description of the laboratory experiments on the fermentation of MEL and the measurement of experimental data can be found in the publication of Beck et al. (2022). The LCA scenarios analyzed in this study either represent the laboratory results described by Beck et al., or are hypothetical projections based on these. The theoretical upscaling for LCA purposes, transferring the experimental laboratory scale to a pilot scale bioreactor with 10 m³ total volume, is based on kinetic models and dimensionless process metrics. A schematic process diagram of the MEL fermentation with relevant parameters for the LCA is shown in Figure 3.

The analyzed system for the base case scenario includes the following process steps. First, a 500 L seed culture of *Moesziomyces* sp. is prepared in a 1-m³-seed reactor by addition of culture medium, glucose, and inoculum. For the production culture in the 10-m³-fermenter, substrates, and culture medium (nutrients, pH buffer and water) are added to a bioreactor and inoculated with the seed culture to a total volume of 5 m³. The fermentation occurs under controlled environmental parameters like stirring, aeration, as well as temperature and pH control, thus consuming electricity, water, steam and air. Sulfuric acid and sodium hydroxide are added during the fermentation to maintain the pH. The fermentation is designed as a fed-batch growth phase with a subsequent production phase, in which the oil substrate is added batch-wise in several steps over the total fermentation duration of 336 h. The final volume of materials in the reactor ultimately amounts to 7.1 m^3^. Additionally, cleaning and sterilization of the reactor and substrates were taken into account, for which the mass and energy consumption are based on calculations and simulations implemented in SuperPro Designer (Oraby et al., 2022). The CO_2_ emissions resulting from the microbial conversion of the substrates in the reactor was also included in the model. An additional inventory list for the presented fermentation can be found as Supplementary Material.

**FIGURE 4:**
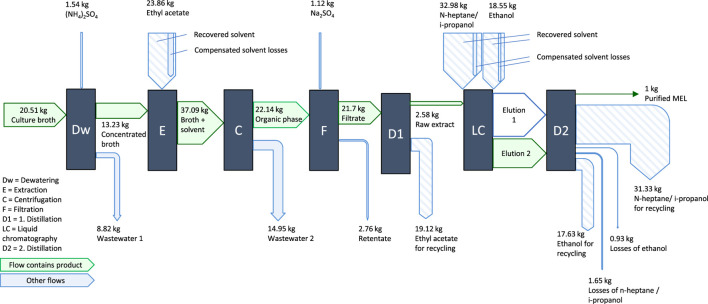
Process steps of MEL purification, mass flows are normalized to the production of 1 kg pure MEL.

The culture broth is then passed on to the downstream purification process, schematically shown in Figure 4. After an initial dewatering step to reduce the processed volume, the purification is carried out as solvent extraction with ethyl acetate followed by flash chromatography with n-heptane, isopropanol and ethanol, as described by (Beck et al., 2019a). For the solvents used, recycling of the solvents by recovery through distillation was assumed with a recycling rate of 95%. Solvent losses by fate in waste streams of processing, e.g., by dissolution in the aqueous phase are considered (Schuster, 2021). The value of 5% for solvent losses is an assumption based on these chemical boundaries and the experience of the laboratory partners. An overview of the process streams during purification is given in Figure 4.

The life cycle inventory (LCI) represents the material and energy flows associated with the production system. To set up the LCI, the software “LCA for Experts (GaBi)” Version 10.7.183 was used for modeling the foreground system and for performing the life cycle inventory analysis (LCIA) (Sphera Solutions GmbH, 2023a). Sphera´s Managed LCA Content (formerly known as GaBi Professional databases, content version 2023.1) provided data for the background system including production of substrates and processes related to material and energy supply used for MEL production (Sphera Solutions GmbH, 2023b). The fermentation was assumed to take place in Germany and, therefore, the German electricity grid mix (21% wind, 19% lignite, 15% natural gas, 12% nuclear, 9% hard coal, 8% photovoltaics, 7% biomass, 5% hydro, 3% other energy sources) for 2019, the most recent available reference year, is considered (Sphera Solutions GmbH, 2023c).

### Chosen impact categories and impact assessment methodology

The impact assessment was performed for selected environmental impact categories following the EF 3.1 impact assessment methods. The EF 3.1 set of methods was selected due to the geographical scope of the LCA, and recent development and recommendation of the EU, and the relevance of the methodology. From this, the following indicators were selected as being sufficiently robust and particularly relevant for the studied product system:

• Acidification,
• Eutrophication, freshwater,
• Eutrophication, marine ecosystems,
• Eutrophication, terrestrial,
• Climate Change, total,
• Ozone Depletion,
• Resource use, fossil, and
• Resource use, minerals and metals.

Additionally, due to particular relevance for analysis of the studied product system, the inventory indicator for primary energy demand from renewable and non-renewable resources, was assessed.

### Process optimization scenarios

The scenario analysis was carried out to identify the potentials for process optimization during the fermentation step and to quantify the effect on the environmental impacts of MEL production. Scenario 3 was defined as the reference scenario, as it provides a basis for experimental improvements accompanied by LCA, and for which a kinetic model is available (Beck et al., 2022). The scenarios were sorted chronologically to show the progress in experimental process development, which was accompanied by LCA and complementary theoretical projections. Here, scenarios 1 and 2 base on a chronologically earlier realized experiment compared to the base case scenario, while others have been performed afterwards. The respective parameters for each scenario are presented in Table 1, with the parameters that vary from the base case scenario FB1-Exp (scenario 3) shown in bold. The descriptions of the scenarios and the corresponding experimental or projective hypothetical changes compared to the base scenario are described in the following section.

**TABLE 1:** Process parameters for scenario analysis, variation of the parameters is based on the identified hotspots for the fermentation. B = batch growth, FB = fed-batch growth, Exp = experimental scenario, Pro = projection, Opt = optimistic projection, renew = renewable energies.

Parameter scenario [with reference no. for experiments according to (Beck et al., 2022)]	No.	Scenario code	Process duration [h]	Power of fermenter [kW/m³*]	Aeration rate [vvm]	Energy demand for compressed air [MJ/Nm³]	Substrate type and quantity (see Figure 3)		MEL yield from oil [g/g]	Concen-tration of MEL/impurities in broth [g/L]
							Glucose [g/L]	Oil [mL oil/L culture broth]		
B2 Experimental	1	B2-Exp	336	**1.0**	0.5	0.3	**30**	Rapeseed, 210	0.20	**29/103**
B Enhanced projection	2	B2-Pro	**240**	0.8	**0.25**	**0.2**	**30**	Rapeseed, 210	**0.35**	**50/100**
FB1 Experimental, base case	3	FB1-Exp	336	0.8	0.5	0.3	65	Rapeseed, 210	0.20	50/83
FB1 Enhanced projection	4	FB1-Pro	**240**	0.8	**0.25**	**0.2**	**90**	Rapeseed, 210	**0.35**	**70/63**
B1 Experimental	5	B1-Exp	**168**	0.8	0.5	0.3	**30**	Rapeseed, **60**	0.20	**10.4/1.9**
FB3 Experimental	6	FB3-Exp	**168**	0.8	0.5	0.3	65	Rapeseed, **120**	**0.39**	**35.2/4.5**
FB3 Soy	7	FB3-Soy	**168**	0.8	0.5	0.3	65	**Soy, 120**	**0.39**	**35.2/4.5**
FB3 Hermetia	8	FB3-Hm	**168**	0.8	0.5	0.3	65	**Hermetia, 120**	**0.39**	**35.2/4.5**
FB3 Enhanced projection	9	FB3-Pro	**168**	**0.6**	**0.25**	**0.2**	65	Rapeseed, **120**	**0.39**	**35.2/4.5**
FB3 Optimistic projection	10	FB3-Opt	**168**	**0.6**	**0.1**	**0.2**	65	Rapeseed, **120**	**0.39**	**35.2/4.5**
FB3 Optimistic projection with renewable energies	11	FB3-Opt-renew	**168**	**0.6**	**0.1**	**0.2**	65	Rapeseed, **120**	**0.39**	**35.2/4.5**

Values in bold represent variations from the base case (scenario 3).

The base case scenario FB1-Exp, which is analyzed in more detail in the contribution analysis, refers to the upscale of data from laboratory experiments, where the fermentation was carried out as a repeated fed-batch fermentation. A glucose fed-batch was used during the growth phase to increase the microbial biomass concentration, which in turn led to a higher productivity during the production phase, where the vegetable oil is converted into MEL. In scenario FB1-Exp, a large amount of oil (21% v/v) was fed to the reactor, which could not be fully converted by the microorganisms, so that at the end of the process a significant amount of impurities, mainly fatty acids, was left.

The first type of alternative scenarios are experimental scenarios (denoted with “-Exp”). They are based on actual experimental changes to the process in the laboratory, e.g., using modified process operation strategies compared to the base case scenario with adjusted feeding schemes and amounts of substrate added. Scenario B2-Exp describes an experimental fermentation chronologically preceding to the base case scenario experiment FB1. It represents a batch growth phase with lower biomass concentration, leading to a lower MEL yield, lower substrate conversion and therefore higher residual substrate lipids compared to the already enhanced base case scenario. The experimental fermentation scenario B1-Exp is also performed with a batch growth phase, but with a reduced oil feed (6% v/v) compared to B2-Exp (21% v/v), in order to achieve a higher substrate conversion and lower concentration of by-products and unconverted substrate, although at the cost of a lower MEL concentration. Finally, the experimental fermentation FB3-Exp combines a glucose fed-batch operation during growth phase to achieve higher biomass concentrations, and an adapted oil feeding (12% v/v) to achieve high substrate conversion and low concentration of unconverted substrate during the MEL production phase, while at the same time maintaining a high MEL concentration. As a result of the increased amount of oil compared to B1-Exp, the scenario shows a higher product concentration in the culture broth. The modified process operations and experimental process optimization approaches for B1, B2, FB1 and FB3 are described in detail by Beck et al. (2022).

Taking these experimental scenarios as a starting point, further theoretical improvements in selected process parameters aim to show the influence of these variable settings on the overall results of the fermentation process. The theoretical projection scenarios for the respective experimental fermentation scenario (denoted with “-Pro”) are defined to assess the implementation of prospective optimizations in the fermentation processes during the development stage and represent optimistic yet realistic assumptions. They represent variations in the fermentation duration, the specific energy demand for stirring and supply of compressed air for aeration, the aeration rate, the oil substrate types and amount, thus influencing the MEL yield and the concentration of impurities in the fermentation broth. Projective scenario for B2-Pro and FB1-Pro assume a reduction of fermentation duration to 240 h, reduced power consumption of the fermenter of 0.8 kW/m³ and 0.2 MJ/Nm³ for compressed air generation, and a reduced aeration rate of 0.25 vvm. The hypothetical enhanced process parameters for each scenario are shown in Table 1 in detail. Moreover, alternative substrate scenarios were defined based on the experimental values of the base scenario FB3-Exp. They consider the use of a different oil substrate instead of rapeseed oil in the base scenario. These are soybean oil and Hermetia oil from the insect larvae of the black soldier fly (*Hermetia illucens*). The latter represents a secondary feedstock that can be obtained from agro-industrial byproducts. The LCI of the production of Hermetia oil is based on upscaled measurement and planning data from a pilot plant of a Hermetia oil producer in Germany (Bippus, 2021; Briem et al., 2021). Larval growth was assumed to occur on Dried Distillers Grains with Solubles (DGSS), a by-product of bioethanol production from wheat. Economic allocation was applied for the feeding substrate DDGS and bioethanol. The LCI for larvae breeding and processing into oil, such as mass flows and energy requirements, are based on feeding trials, upscaling using dimensionless process parameters, and planning data for equipment of an industrial rearing facility. Mass allocation is applied to Hermetia oil and its co-product protein meal. In an experimental fermentation series described by Beck et al. (2019a), the researchers investigated the influence of plant oils on MEL production and showed, that substitution of the oil substrate does not significantly change the yield and structure of MEL. For this reason, the MEL yield was assumed to be constant for the scenarios with substitution of described oil substrates. Though minor structural changes in the biosurfactant side chains may occur depending on the oil used, it was shown that the structure of the biosurfactants produced is mainly determined by the producing organisms and less by the substrates (Beck et al., 2019a).

Ultimately, the optimistic scenarios (denoted with “-Opt”) are based on further enhanced hypothetical but realistic assumptions for aeration rates, energy efficiency of equipment and increased product yields. They thus provide an outlook for evaluating the potentials for optimization in the future. The parameter values for the respective lower aeration rates, energy requirements for fermenter and compressed air supply, as well as the assumed improved MEL yield are given in Table 1.

### Kinetic LCA model

A kinetic model of the fermentation process for MEL production was previously developed based on experimental data measured in the bioreactor, which was then fitted to Monod kinetics to simulate the conditions in the bioreactor over the process duration and to estimate the substrate turnover and product concentration (Beck et al., 2022). The kinetic model for the MEL production phase is described by ordinary differential equations. Numerical solutions for substrate and product concentrations were implemented on an explicit Euler method in Microsoft Excel. The complete details of the model equations and input parameters can be found in Beck et al. (2022). The idea of combining the kinetic model with LCA results allows further analysis beyond the options provided by scenario analysis. This makes it possible to derive a method for calculating progressions and optima of time-dependent process parameters and environmental impacts under consideration of substrate conversion and product formation dynamics. It is expected that this approach will allow to determine, e.g., an improved understanding for the optimal fermentation time under certain boundary conditions. Taking the time course of substrate and product concentrations from the Monod kinetics model as the starting point, a kinetic LCA model was developed in this work. In the kinetic LCA model, process conditions over time are considered in 0.2-h steps to calculate LCA results over the process duration. This includes the respective concentrations of MEL, concentrations of unconverted substrate, the amounts of oil substrates, and energy consumed for stirring and aeration for the fermentation process up to each point in time. On the input side, the preculture and fed substrates are added cumulatively up to the respective analysis time. The impacts proportional to the fermentation time, e.g., energy consumption for agitation and aeration, are scaled over the runtime of the production culture from the static result. This way, a life cycle inventory is calculated for each point in time over the fermentation duration, with the respective substrate and energy inputs and MEL output. The kinetic LCA analysis is carried out for the experimental scenarios FB1-Exp and B2-Exp. To scale the results to 1 kg MEL for each point in time, first the impacts of substrates and energy required up to that point are calculated, and then scaled with the current amount of MEL produced in the fermenter up to this point. Thus, the results of the kinetic LCA analysis are normalized to the “static end point” of each experiment, which is defined at 100%, to visualize the process optimization potentials at each point in time.

## Results

### LCIA contribution analysis for the base case scenario

**FIGURE 5:**
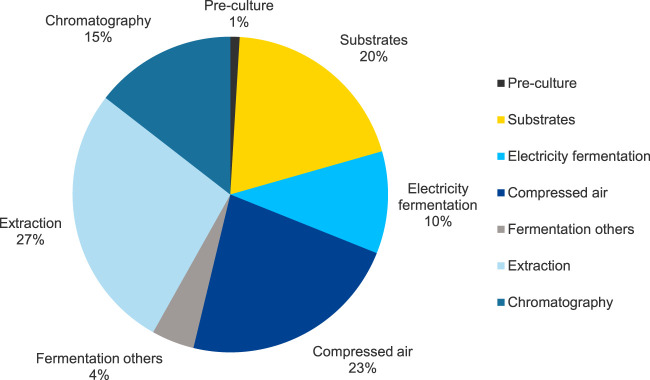
Results of the contribution analysis for the fermentation and purification processes for MEL production for base case scenario FB1-Exp (scenario 3) in the impact category EF 3.1 Climate Change, total.

The results of the contribution analysis of the MEL production, including fermentation and purification, for base case scenario FB1-Exp in the impact category Climate Change are shown in Figure 5. Overall, the fermentation section contributes to about 58% and the purification section to 42% of the total production impact.

The processes for operating the bioreactor, specifically aeration and stirring, as well as the provision of substrates, have the highest contribution to the fermentation and are therefore identified as hotspots. The environmental impacts for the operation of the reactor can be attributed to the generation of electrical energy for aeration and stirring, which together account for about 33% of the total impact in the category Climate Change. More precisely, about 23% is caused by electricity consumption for generating pressurized air for the aeration of the reactor, and about 10% is attributed to providing electricity for other equipment to run the reactor, i.e., mainly agitation. In terms of feedstock, a large share of about 20% of the potential environmental impact on Climate Change is related to substrates, mainly the provision of vegetable oil (15%) and, to a lesser extent, glucose (5%). These impacts are primarily caused by the cultivation of the feedstock crops, in this case rapeseed, e.g., through emissions from machine use during cultivation, and the production and application of fertilizers. The processing of rapeseed into vegetable oil also contributes to a certain extent. The preculture and the components of the culture medium have a minor influence in this impact category and account only for approximately 1% of the overall result. Other processes contribute to 4% of the impacts in this category. These include, for example, media sterilization, cleaning-in-place and sterilization-in-place of the reactor, and supply of cooling water for the bioreactor.

The purification, consisting of a solvent extraction followed by flash chromatography, significantly contributes to the potential impacts of the overall MEL production in the category Climate Change. The purification steps account for about 42% of the total impact in the category Climate Change in the cradle-to-gate analysis of MEL production, of which 27% are attributed to the extraction step with ethyl acetate, and 15% for the following flash chromatography. The use of solvents for both extraction and chromatography has a noticeable influence on the potential environmental impacts of the purification. Although the solvent recycling rate was assumed to be at 95%, the provision of 5% fresh solvents to compensate the inevitable loss of solvents, e.g., through dissolution in the aqueous phase during extraction, contributes significantly to the potential environmental impacts. The provision of thermal energy for evaporating the solvents during distillation also has a significant impact. Other operating materials used in the purification steps, as well as electricity used for the agitation of stirred reactors for mixing during the extraction, have a comparatively low contribution to the impacts on Climate Change.

**FIGURE 6:**
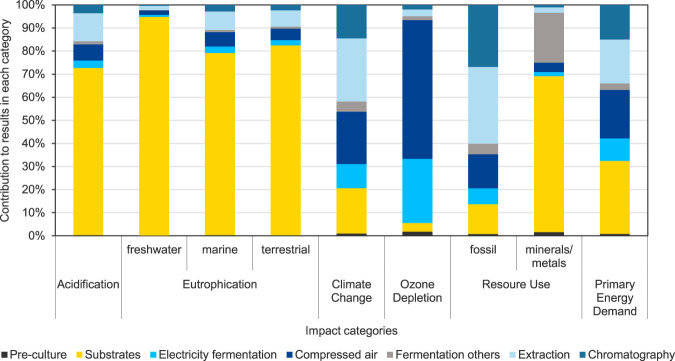
Results of the contribution analysis for the fermentation and purification processes for MEL production for base case scenario FB1-Exp (scenario 3) for selected EF 3.1 impact assessment categories and primary energy demand.

Figure 6 shows the contribution analysis for selected impact categories in addition to the previously discussed impact category Climate Change. It can be seen that the different process steps have differing relative impacts, depending on the respective impact category. In the impact category Acidification (AP), and the three categories Eutrophication (EP) freshwater, marine, and terrestrial, the main contributions are linked to the provision of substrates for MEL fermentation. This is due to the agricultural processes used to cultivate the crops, such as the use of fertilizers. The contribution to Climate Change is composed of a variety of processes and steps and has already been presented in detail in the previous section. The impacts in the category Ozone Depletion are mainly attributed to electricity consumption and thus the electricity-intensive processes of providing compressed air for aeration and stirring of the fermenter contribute significantly. In the category Resource Use, minerals and metals, the substrates are identified as the main contributor as well. These impacts are also attributable to the agricultural processes to provide the substrates for the impacts. The purification steps contribute most to the impact category Resource Use, fossil due to the solvent use, but to some extent also due to the energy consumption of energy-intensive process steps in the purification section, such as distillation. With regard to the Primary Energy Demand (PED), not only the substrates, but also the compressed air generation, i.e., the underlying electricity, are relevant processes in the fermentation of MEL. For the purification, about one-third of the impacts of MEL production on PED is attributable to extraction and chromatography steps. These are mainly attributed to the compensation of solvent losses and the energy-intensive processing steps, such as solvent evaporation and the corresponding thermal energy consumption. Hence, the different process steps for MEL production are of varying importance in each of impact categories considered. While the impacts for AP, EP, and Resource Use, minerals and metals, are dominated by the agricultural cultivation processes for substrates, the production of solvents and the provision of electrical and thermal energy are essential for the potential impacts in the other impact categories.

### LCIA results for process optimization scenarios

**FIGURE 7:**
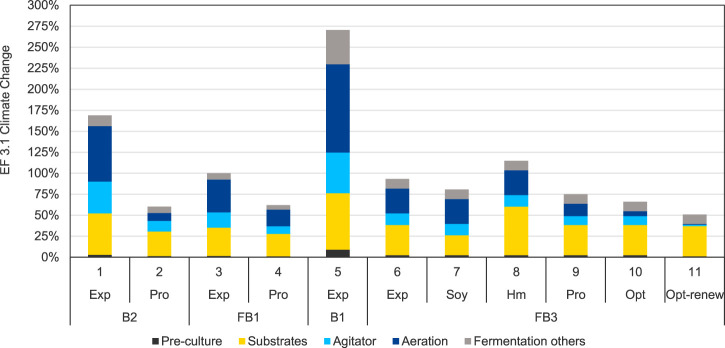
Scenario analysis for process optimization scenarios for the fermentation and purification processes for MEL production in the impact category EF 3.1 Climate Change, total, impacts are normalized to the base case scenario FB1-Exp (scenario 3).

The scenario analysis for optimization strategies described in this section, focusses on the fermentation process only, while purification is not considered further. Since many different purification processes are conceivable, which strongly depend on the composition of the broth at the end of the fermentation, a scenario analysis for purification, especially in connection with different fermentation scenarios, would go beyond the scope of this work. The topic of applying LCA to biosurfactants purification optimization is subject of continuing research and therefore future publication. In contrast, the aim of this scenario analysis is the identification of optimization options only for the fermentation process, without the restriction to combine the optimized fermentation processes with the previously investigated purification route of solvent extraction and chromatography of the base case. The presented scenarios depict the potential of various realized process improvement measures in the bioreactor with regards to their environmental benefits. At the same time, they also reflect the progress of the development work in the laboratory. In addition to the scenarios based on experimental data, several hypothetical prospective scenarios, in which some parameter variations are based on optimistic but realistic assumptions, show the optimization potential of intermediate or future process improvements. The scenario analysis generally shows that the variation of the product yield, the duration of the fermentation, the aeration rate, the energy efficiency of equipment used, e.g., compressors and agitation, significantly influences the results of the LCA. For both the hypothetical and the experimentally realized scenarios, product yield is identified as the most important parameter, as the product quantity per batch scales the impacts of all material and energy inputs. The results of the scenario analysis in the category Climate Change are presented in Figure 7. Scenario 3 (FB1-Exp) represents the base case scenario and is accordingly scaled to 100%. The results of the scenario analysis are presented for the impact category Climate Change.

Scenario 1 (B2-Exp) is the chronologically first of the investigated scenarios and represents the upscaled experimental laboratory data for experiment B2. A batch growth phase followed by repeated oil addition was modelled here, leading to a MEL concentration of 29 g/L with a very high residual impurities concentration. Aeration, electricity consumption of the fermenter, and substrates provision were identified as main contributions. Based on those findings, a hypothetic but realistic projection was drawn to investigate prospective improvements, resulting in scenario 2 (B2-Pro). The optimistic projection B2-Pro shows significant impact reductions in all fermentation process steps. The reductions in the optimistic projection scenarios can mostly be seen in the process steps dominated by energy demand compared to the respective experimental scenario and demonstrates the significant potential for environmental improvement associated with these process steps.

With this analysis of the hypothetical projection of the first experiment B2, the first experimental optimization activities in the laboratory to improve the fermentation process are implemented in experiment FB1, represented by scenario 3. The first optimization activities in the laboratory, like implementing a fed-batch growth phase for higher biomass concentration and thus higher productivity, leading to a higher MEL concentration of around 50 g/L, have shown to already reduce the impacts of scenario 3 (FB1-Exp) compared to the early laboratory state of scenario 1 (B2-Exp) by about 40%.

Again, hypothetical process improvements, which are mostly based on more efficient stirring and aeration and a reduced fermentation time, were then defined for experiment FB1, represented in scenario 4 (FB1-Pro). With the hypothetical assumptions analogously of B2-Pro, Scenario 4 also shows significant reductions compared to the corresponding experimental scenario 3. The difference between the experimental and the projective scenario is smaller here than in for B2 (scenario 1 and 2), as significant optimizations have already been implemented in the respective experimental reference scenario FB1.

In experimental fermentation B1 represented with scenario 5 (B1-Exp), a batch growth fermentation with a single oil feed was applied with the goal of converting the substrate as completely as possible while having the lowest possible amounts of impurities in the culture broth. Due to this focus in the process design, which is characterized by a lower substrate addition, a lower product concentration of only 10.4 g/L was achieved. The impacts per kg of pure MEL due to the processes for operating the reactor, as well as the process steps for the preculture and substrate to the grow biomass, are particularly high in this case compared to the other scenarios. This underlines the importance of the product yield per batch as an important process optimization parameter. However, this process design could have advantages if the high purity allows a simplified purification for specific MEL applications. However, this still needs to be determined in further experiments and environmental assessment.

The experiment for scenario 6 (FB3-Exp) represents the most developed laboratory development status from the experimental perspective described by Beck et al. (2022). The experimental improvements, e.g., a higher product concentration in the culture broth, but also the shorter fermentation duration through fed-batch growth and thus higher biomass concentration lead to a reduction of environmental impacts. On the one hand, this is due to the higher product quantity per batch, which means that the impact of the required inputs per kg MEL is respectively lower. On the other hand, the energy-related impacts are reduced due to the shorter operating time of the reactor and equipment.

In the alternative substrate scenarios 7 and 8 (FB3-Soy and FB3-Hm), it is assumed that a different oil source is used as substrate instead of rapeseed oil. In scenario 7 with soy oil, a reduction in the potential impacts in the category Climate Change of 13% to scenario 6 (FB3-Exp) can be seen due to the substitution of rapeseed oil. In the scenario with Hermetia oil, which is based on an LCA model of an upscaled pilot plant for Hermetia rearing and processing, 23% higher impacts in the category Climate Changeare attributed to MEL production due to the oil substrate substitution. Here, further optimizations in rearing and scale effects offer potentials to reduce the impact of Hermetia oil production in the future and potentially offer an alternative oil source based on secondary raw materials. Overall, the oil substitution scenarios demonstrate that the change of substrates can lead to significant variations in environmental performance. For this reason, the investigation of secondary raw materials as substrates for bioprocess production provides a strategy to reduce impacts by using more environmentally advantageous feedstocks. Scenarios 9, 10 and 11 give an optimistic yet realistic outlook on the experimentally achieved conditions of FB3-Exp. Through hypothetical optimistic adaptions and optimized process assumptions, such as a reduced and demand-oriented optimization and agitation, a reduction of the impact by 34% for scenario 10 (Fb3-Opt) compared to the base case would be achieved. If additionally, electricity from renewable sources was used for the operation of the fermenter, the impact could even be reduced by 49% in scenario 11 (FB3-Opt-renew) compared to the base case scenario. In this case, if the energy-intensive process-related impacts of the fermentation are assumed to be largely optimized, mainly the substrates would contribute to the impacts of the fermentation of MEL. Consequently, the use of secondary materials would become increasingly relevant.

### Kinetic LCIA results for fermentation

**FIGURE 8:**
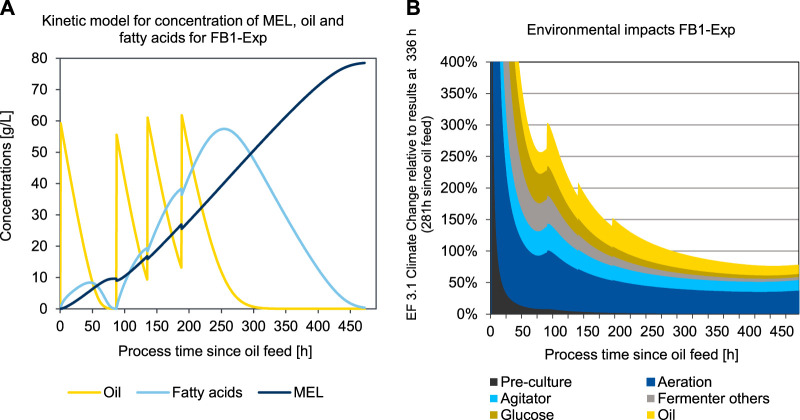
Concentration of oil, fatty acids, and MEL **(A)** and kinetic presentation of LCA results in the impact category EF 3.1 Climate Change, total **(B)** over process duration of the production culture for a fed-batch fermentation of MEL using a kinetic substrate conversion model for FB1-Exp (scenario 3).

To determine the time course of the environmental impacts during the fermentation process, a kinetic LCA model was developed and applied for two different scenarios, the base case scenario 3 (FB1-Exp) and the enhanced experimental scenario 5 (FB3-Exp). The kinetic LCA results for the base case scenario 3 (FB1-Exp) are shown in Figure 8.

Figure 8A shows the concentration profiles of oil added to the fermenter, as well as the concentration of fatty acids as an intermediate product, and the concentration of MEL formed over the process time since the first oil feed (in scenario FB1-Exp after 55 h). This point represents the transition from the growth phase to the production phase and is thus set as the origin of the time axis in Figures 8A, B. Figure 8B shows the resulting impacts in the category EF 3.1 Climate Change attributed to 1 kg of MEL, if the fermentation was to be stopped at the corresponding time. Thus, all fermentation impacts that have occurred up to the respective point in time are scaled to the amount of product in the broth at that point in time. At the beginning of the MEL production phase, the environmental impacts are asymptotically infinite, since at this time there is no product yet and therefore the impacts per batch are divided by a small amount of product.

The initially high environmental impacts at the beginning of the production phase (resulting from pre-culture and addition of glucose in the previous growth phase) become continuously lower as the MEL concentration increases. This is reflected in the graph in Figure 8B with a continuous decrease of the specific environmental impacts of the pre-culture and growth phase over time. Since the process is designed as a fed-batch fermentation with four oil feedings, four peaks for the impacts of batch-wise added oil input can be seen accordingly whenever fresh oil is added to the fermenter. On the other hand, the environmental impacts of aeration and agitation increase linearly over time in relation to the batch. However, by scaling the impacts to the product quantity, hence taking into account the specific impacts for 1 kg of MEL, these impacts initially decrease as the product quantity increase outweighs the effect of additional electricity consumption. When in the further progression of the fermentation the product formation rate decreases, and the additional product quantity formed slows down, then the impacts for electricity use approaches a plateau. At the point, where the effect of additional product formation no longer exceeds the impacts of additional impacts for electricity consumption, the optimal fermentation duration is reached. For the case illustrated in Figure 8B, this minimum is reached after 437 h in the production phase, which equals a total fermentation duration of 492 h in the production reactor.

**FIGURE 9:**
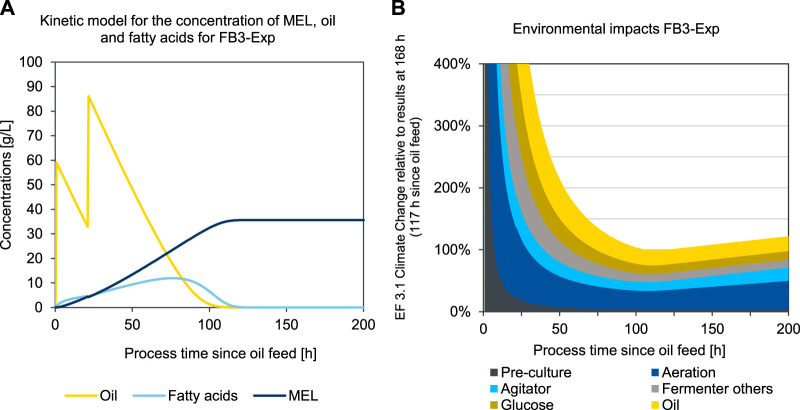
Concentration of oil, fatty acids, and MEL **(A)** and kinetic presentation of LCA results in the impact category EF 3.1 Climate Change, total **(B)** over process duration of the production culture for an optimized fed-batch fermentation of MEL using a kinetic substrate conversion model for FB3-Exp (scenario 6).

The second case, illustrated in Figures 9A, B, represents the optimized experiment FB3-Exp (scenario 6) For this second case, a fed-batch experiment with only two oil feeds is considered, in order to shorten the process time and to fully convert the substrate. The production phase is started with the first oil feed after a growth phase of 51 h, which is again set as the origin of time axis in Figures 9A, B. In this experiment, the process was continued for a longer time after the substrate was fully consumed to obtain a sufficiently long time series for concentrations (compare Figure 9A), and to ensure that the optimum fermentation time was clearly exceeded to visualize the progression after the environmental minimum (compare Figure 9B). The qualitative curve of the specific impacts per product quantity is analogous to the description in the above examined experiment. Due to adapted oil feeding, however, the substrate is consumed earlier, and MEL formation stagnates accordingly. Therefore, the environmental minimum is also reached earlier, after 113 h in the production phase, which equals a total process duration of 164 h in the production reactor. Due to the changes in the process parameters in FB3-Exp, the substrate is used up much earlier in this case and the environmental minimum is therefore reached earlier compared to FB1-Exp. After a plateau in the area of the minimum, the impacts then rise again almost linearly, due to the continuous power consumption for operating the bioreactor, without any additional MEL being produced after the substrate has been used up. Hence, the point of minimum environmental impact could be clearly determined, as visible in Figure 9B.

## Discussion

### Data quality

The overall data quality is considered high regarding technical representativeness. For most datasets, country-specific and temporally up-to-date datasets are used that represent the current situation for MEL production in Germany at the present time well. Some uncertainties may derive from assumptions and upscaling of laboratory processes compared to the use of industrial primary data for purification. Despite these potential uncertainties associated with the LCA results, the relevant process steps for fermentation optimization can be identified. The methodological approach for determining the optimal time to terminate a fermentation using environmental indicators is based on kinetic models. The underlying Michaelis-Menten kinetics matches well with the measured experimental data and seems to describe the culture behavior sufficiently accurately to be used for this purpose.

### Comparison with results of other biosurfactant LCA studies

The general results and trends are found to be in line with the few existing LCA studies on biosurfactant production, while certain variations occur due to the different product systems, as well as different methods applied. A shared finding with Kopsahelis et al.‘s study on LCA of sophorolipid and rhamnolipid production is that substrates have a significant contribution to the overall results in many impact categories (Kopsahelis et al., 2018). The fermentation processes have also been shown in other studies to make a significant contribution in the category Climate Change. Kopsahelis et al. (2018) found considerable differences in the environmental impacts of the two biosurfactants, due to the different fermentation duration. This aligns with the influence of fermentation duration on the results of the scenario analysis for MEL fermentation in this case. As far as purification was considered, the downstream processing of the culture broth was also identified as relevant for environmental sustainability (Baccile et al., 2017). However, the specific processes of biosurfactant purification with extraction and flash chromatography considered in our study, have not yet been considered in other published and reviewed LCA studies so far.

For the environmental evaluation of biotechnological processes, Lima-Ramos et al. and Meissner and Woodley propose a set of indicators and metrics to use in process development (Lima-Ramos et al., 2014; Meissner and Woodley, 2022). However, Lima-Ramos et al. do not recommend using LCA-based indicators for evaluation at an early stage due to not yet fully defined and optimized process, but at a late development stage (Lima-Ramos et al., 2014). The general questions of at what point in process development LCA should be carried out and in what way, is currently subject of research activities in the LCA community. Although there is usually few data available at an early stage of development, and therefore setting up an inventory is more complex and the results less accurate, the degrees of freedom for eco-design can still be exploited to the maximum in the early product development stages. In general, dimensionless process metrics can be used to simulate an upscaled process and many environmentally beneficial scale effects can be integrated into early-stage sustainability assessment this way. However, mature processes tend to have lower environmental impact due to decades of optimized efficiency, integrated systems, and synergies with other processes or sectors compared to laboratory or pilot-scale bioprocesses. These can often not be addressed adequately at an early stage, which practically hinders comparisons to established processes, but reveal optimization potentials and allow to derive further optimization strategies. In our case, the detailed investigation of specific environmental aspects of process design using LCA could be achieved at a quite early development stage in close collaboration with the laboratory process development and the respective upscaling models.

### Identified fields of action for process optimization for MEL fermentation

This study shows that both fermentation and downstream processing make an important contribution to the environmental impacts of MEL production. The LCA results point out the opportunities to reduce environmental impacts through various possible process optimization options. Increased yield during fermentation strongly affects the LCA results, as the amount of MEL produced scales the impacts assigned per product quantity. Aeration causes high impacts in the impact category Climate Change and Ozone depletion, due to the electricity use for the compressed air supply, especially for the scenarios representing the early stages of development of MEL fermentation. The results of the scenario analysis with optimistic projections indicate that the major potentials for reducing the impact of aeration are, on the one hand, the introduction of a demand-based air supply. Advanced sensor systems like dissolved oxygen measurement and off-gas analysis may help to determine the oxygen demand according to the current state of the cells and to supply only the required amount of air to the reactor (Beck et al., 2022). In addition, the use of renewable energies for the operation of the bioreactor holds considerable savings potential regarding greenhouse gas emission reduction. Moreover, a decrease in process duration results in a further potential for lowering the impacts due to aeration and agitation. This can be achieved by an increased space-time yield, so that the conversion of the substrate to product can occur in a shorter time.

Substrate provision is also a major source of environmental impacts in the fermentation in several impact categories. Therefore, the use of low impact substrates can reduce environmental impacts significantly. Especially after considering the process-related optimizations for fermentation conditions like aeration and stirring, the cultivation and provision of oil substrates, such as rapeseed oil, remains the most relevant process in the fermentation relevant impact categories. Regarding alternative oil substrate sources, the results show that different vegetable oils suitable for MEL production with specific environmental profiles affect the results in a range of 13% lower to 23% higher impacts for MEL production for the assessed oil substitutions, and therefore offer an opportunity to reduce the impact of the MEL through appropriate selection. Despite the higher impact of Hermetia oil in the impact category Climate Change relative to rapeseed oil the comparability here is limited due to the development stage and the production scaling of Hermetia oil, as described in the materials section. The valorization of low cost and low impact second and third generation feedstock that do not directly compete with food crops, e.g., agro-industrial by-products and residues, will be necessary to lowering the impacts from substrate provision for biosurfactants. Mohanty et al. (2021) and Miao et al. (2024) provide an overview over various secondary feedstock for biosurfactants and discuss the challenges of raw material availability, large scale production, and requirement of pre-treatments and downstream processing. Since second and third generation feedstocks have been studied more extensively with LCA for the production of biofuels than for the production of biosurfactants, findings and methodological approaches could also be transferred, e.g., regarding allocation approaches. The results based on the not yet fully optimized production processes indicate that alternative oil sources should also be considered as an opportunity to reduce the impact from substrate provision and should therefore be further investigated.

In addition, the purification was identified as an important section of the MEL production and, in particular, solvent use as the most important factor influencing the environmental impacts of the purification. Therefore, in further research and development activities for MEL, the substitution of the currently used solvents with environmentally friendly alternatives, or the use of a different purification process, need to be investigated. The sensitivity of the assumption for the solvent recovery rate is likely high, however, as this article focuses on the fermentation process and optimization, this has not been investigated further in this study and can be considered a limitation. However, future analyses should investigate this further and in context with the purification steps and their optimization potentials. Consequently, purification optimizations should also be evaluated with LCA simultaneously to the experimental process development, to identify the most environmentally optimized option at an early stage. The developed LCA model for fermentation of purification route of this case will serve as a good base for these investigations.

Additionally, the progress made in the laboratory process development could be tracked and presented with LCA, while projections serve as outlook for future optimization potentials and guide process development. Here, especially the scenario analysis serves as basis for accompanying the experimental adaptions made to the laboratory process and supporting possible future developments to increase the environmental performance of biosurfactants. This way, LCA contributes to the monitoring of progress and ensures that process improvements are accompanied by improved environmental performance. Additionally, when moving from laboratory to industrial scale, environmental advantages from upscaling can be expected. Furthermore, integrated systems with energy recovery will then become more feasible, leading to further energy efficiency improvements for MEL production. However, as these are very plant-specific and difficult to estimate with sufficiently high accuracy at the current development stage, they are therefore not assessed for this case of early-stage process optimization.

In general, changes in process control that lead to an increase in yield but involve additional energy or material input should always be evaluated to balance the additional environmental impacts with the savings. As a relevant example, fermentation and DSP cannot be considered isolated from one another, because fermentation parameters influence the culture broth to be purified. Therefore, the coupling of the relevant fermentation parameters to the DSP parameters can provide further valuable findings. A detailed system analysis for this interaction using LCA will support addressing the issue of how to find the environmentally most favorable combination of fermentation and DSP variants and parameters and must hence be tackled in the future to enable the production of environmentally sustainable biosurfactants.

### LCA as a tool in biotechnological process development

LCA can both be used to assess optimization potentials by using projections, but also to quantify the experimental improvements to the process. The results show that optimization potentials identified in the LCA can be used for experimental process optimization in the laboratory, and that the progress of experimental process development can be tracked with LCA to evaluate process design options from an environmental perspective. It can also be noted that the experimental fermentation scenarios reflect the chronological progression of the process optimizations implemented to the MEL fermentation process in the laboratory. Although the optimistic projections B2-Pro did not have a measured value basis for the assumptions made, they represented optimistic assumptions and targets, of which, e.g., the assumed MEL concentration was achieved in the follow-up experiment FB1 (FB1-Exp). The same applies for the projection of the respective experiment, FB1-Pro, which could mostly be reached or even surpassed by the following experimental scenario FB3. On the one hand, the scenario analysis provides many valuable insights for the system analysis. On the other hand, however, LCA can also be used to address very specific problems for process optimization by combining it with additional domain-specific models, as it was shown with the combination of LCA results with kinetic models. LCA usually provides static results that apply precisely to a defined state. However, biotechnological processes are dynamic processes. In the case of fermentation in particular, the fermentation time has proven to be a sensitive parameter for the environmental impacts. Based on time resolved experimental data, such as information on the concentration of MEL and impurities in the reactor and substrate inputs and energy inputs over time. This provides the possibility of finding environmental optima for specific process parameters, such as in this case the fermentation duration of the MEL production phase. For the case of MEL fermentation, it is demonstrated that the process conditions, such as the optimal process duration to terminate a fermentation, can be determined based on environmental, LCA-based indicators. With the novel kinetic presentation of the LCA results it is shown that there is a plateau around the optimal duration with an almost similar minimum environmental impact. This point in time coincides with the measurable stagnation of the product concentration or with the consumption of the substrate and intermediate product in the example of MEL fermentation. Moreover, process-specific variables, such as the feeding scheme, but also site-specific conditions, such as the consideration of a certain electricity mix, can be considered with this approach. Summing up, this approach provides a novel way to calculate an optimal fermentation time based on an environmental indicator, instead of the usual estimation via process-related, but non-environmental measures. With measurements of substance concentrations alone, this system analysis would not have been possible in terms of environmental sustainability.

The environmental assessment of the particularly detailed process data at this stage of development goes beyond the state-of-the-art assessment of bioprocesses in early development stages. By the close cooperation with the partners’ data collected from experimental and bioprocess modeling, the LCA consequently provides better knowledge on the process chain through its depth of detail, as well as understanding the relevance and improvement potential for individual steps of the process chain. The assessment of several impact categories generates versatile environmental data. It allows a differentiated analysis of the production system and makes tradeoffs between indicators visible. Furthermore, a wider range of LCA cases will help to establish LCA in the development of biosurfactans, as data for individual process steps of biosurfactant production can be used in a modular way. Therefore, this case of LCA of MEL production also contributes to it. Also, the establishment of dedicated process libraries for bioprocesses enables LCAs to be carried out more efficiently. The LCA models and process libraries can be used for further research issues and transferred to related (bio)processes. In addition, process engineers will be informed about the environmental consequences of their optimizing activities, which helps them to optimize their processes efficiently and unlocking the full potential of early-stage evaluations. Besides, with more detailed LCAs on biosurfactants being carried out, the better process developers and engineers know what kind of data is necessary for a meaningful LCA, and what kind of results can be obtained. Altogether, this LCA study demonstrates the potential for further improvements and supports development towards environmentally friendly produced biosurfactants.

## Conclusion

This detailed LCA study on MEL fermentation provides a valuable system analysis for a bioprocess and hence useful information for process engineers to support their decisions. The results and interpretation highlight the important parameters to address for process optimizations from an environmental perspective. The optimization potentials for both experimental and projective process adaptations are assessed, and the improvement potential is determined for the individual steps in the process chain and for each scenario. Hence, the most important process steps and field of action for environmental sustainability optimization can be determined.

The combination of LCA with kinetic models makes it possible to find by quantitative solutions for specific problems of bioprocess design, e.g., for determining the environmentally optimal fermentation duration. This novel kinetic presentation of LCA results can be used to calculate the optimal time to stop fermentation in terms of environmental impact, which had to be done based on other, non-environmental indicators previously. Moreover, this representation enables a better understanding of the influence of this parameter, as the course also makes the plateau of the specific environmental impact visible and thus comprehensible.

Further development potentials for MEL optimization models include the combination of LCA models for fermentation with DSP, which has turned out to be an important part of the process for environmental optimization. The combination of fermentation and DSP for dynamic analysis would be very beneficial in this case. Optimizations in fermentation aimed at simplifying purification can only be environmentally assessed to a limited extent in this case studied. Adjustments to the fermentation are only assessed selectively, but not as a continuous representation of fermentation parameter variations. This could be achieved by specifically extending the LCA model by including the interdependencies of fermentation and DSP and would provide process developers with additional information on the overall process. In the future, combining this approach with data from inline measurements or models used for process automation could be a further development of sustainability assessment for process optimization. Automatically generated or collected data could be co-used for environmental optimization to streamline data collection and setting up fermentation models. In addition, combining process simulation software with LCA could be a further step to providing environmental information for process design decisions more efficiently. The approach of combining LCA with kinetic models could also be transferred to economic models. Vice versa, output quantities from economic process models could be used for further LCA considerations or eco-efficiency analysis. In conclusion, this work represents a detailed environmental analysis of the bioprocess for MEL production and purification, that should be used in further research to support and guide the development of sustainable biosurfactants.

## Data Availability

The datasets presented in this article are not readily available because of confidentiality. Requests to access the datasets should be directed to LB, lars.bippus@iabp.uni-stuttgart.de.
