# Supplementary material for: Life cycle assessment for early-stage process optimization of microbial biosurfactant production using kinetic models—a case study on mannosylerythritol lipids (MEL)
Source: Front Bioeng Biotechnol. 2024 Feb 23;12:1347452. doi: 10.3389/fbioe.2024.1347452 (PMC10920258; doi:10.3389/fbioe.2024.1347452)
Supplement: Supplementary file 1 [file DataSheet1.PDF]

Supplementary information to

Life Cycle Assessment for early-stage process optimization of microbial biosurfactant production using kinetic models – a case study on Mannosylerythritol lipids (MEL)

Lars Bippus[1*], Ann-Kathrin Briem[1,2], Alexander Beck[3,4], Susanne Zibek[3,4], Stefan Albrecht[2]

[1]University of Stuttgart, Institute for Acoustics and Building Physics IABP, Department Life Cycle Engineering GaBi, Stuttgart, Germany

[2]Fraunhofer Institute for Building Physics IBP, Department Life Cycle Engineering GaBi, Stuttgart, Germany

[3]Fraunhofer Institute for Interfacial Engineering and Biotechnology IGB, Stuttgart, Germany

[4]University of Stuttgart, Institute for Interfacial Process Engineering and Plasma Technology IGVP, Stuttgart, Germany

*** Correspondence:**
Corresponding Author
lars.bippus@iabp.uni-stuttgart.de

Table 1: Additional information on the LCI for MEL fermentation base case (FB1-Exp) per batch (10 m^3 reactor volume)

Inventory	Amount and specification	Reference
Glucose	325 kg glucose from corn starch	Values obtained from experiments and process simulation (Beck et al., 2022)
Oil substrate	1050 kg rapeseed oil	Values obtained from experiments and process simulation (Beck et al., 2022)
Pre-culture	500 kg culture suspension	Values obtained from experiments and process simulation (Beck et al., 2022)
Culture medium	4675 kg mineral medium and water	Values obtained from experiments and process simulation (Beck et al., 2022)
pH regulation	150 kg sulfuric acid (1 M)<br>400 kg sodium hydroxide (2 M)	Values obtained from experiments and process simulation (Beck et al., 2022)

Aeration	201.6 nm^3 compressed air (6 bar)	Values obtained from experiments and process simulation (Beck et al., 2022)
Stirring	2177.28 MJ electric energy	Values obtained from experiments and process simulation (Beck et al., 2022)
Cooling water supply	201 MJ energy pumping energy<br>73.9 kg replacement of cooling water losses	Values obtained on equations for energy consumption of average cooling water supply
Continuous media sterilization	1991.2 MJ process steam from natural gas	Values obtained from the SuperPro Designer simulated process (Oraby et al., 2022)
Gaseous carbon emissions	284 kg carbon dioxide (biogenic)	Values obtained from exhaust gas measurements and mass balances (Beck et al., 2022)
Fermenter SIP (Sterilization-In-Place)	500 kg low pressure steam from natural gas<br>1.9 MJ electric energy	Values obtained from the SuperPro Designer simulated process (Oraby et al., 2022)
Fermenter CIP (Cleaning-in-Place)	2000 kg deionized water<br>1000 kg sodium hydroxide solution (2 %)<br>95.4 kg low pressure steam from natural gas<br>17.4 MJ electric energy<br>1.82 nm^3 compressed air (6 bar)<br>25.9 kg sulfuric acid (96 %)<br>174 kg municipal wastewater (carbon content corrected)	Values obtained from the SuperPro Designer simulated process Oraby et al. (2022)

References

Beck, A., Vogt, F., Hägele, L., Rupp, S., and Zibek, S. (2022). Optimization and Kinetic Modeling of a Fed-Batch Fermentation for Mannosylerythritol Lipids (MEL) Production With Moesziomyces aphidis. *Front Bioeng Biotechnol* 10, 913362. doi: 10.3389/fbioe.2022.913362

Oraby, A., Rupp, S., and Zibek, S. (2022). Techno-Economic Analysis as a Driver for Optimisation of Cellobiose Lipid Fermentation and Purification. *Front Bioeng Biotechnol* 10, 913351. doi: 10.3389/fbioe.2022.913351
